# TLR2 modulates inflammation in zymosan-induced arthritis in mice

**DOI:** 10.1186/ar1494

**Published:** 2005-01-21

**Authors:** Matthias E Frasnelli, David Tarussio, Veronique Chobaz-Péclat, Nathalie Busso, Alexander So

**Affiliations:** 1 Laboratoire de Rhumatologie, Centre Hospitalier Universitaire Vaudois, Lausanne, Switzerland

**Keywords:** chronic inflammation, immune system, monocytes/macrophages, Toll-like receptor

## Abstract

The interplay between the innate and acquired immune systems in chronic inflammation is not well documented. We have investigated the mechanisms of inflammation in murine zymosan-induced arthritis (ZIA) in the light of recent data on the roles of Toll-like receptor 2 (TLR2) and Dectin-1 in the activation of monocyte/macrophages by zymosan. The severity of inflammation, joint histology, lymphocyte proliferation and antibody production in response to zymosan were analyzed in mice deficient in TLR2 and complement C3, and the effects of Dectin-1 inhibition by laminarin were studied. In comparison with wild-type animals, TLR2-deficient mice showed a significant decrease in the early (day 1) and late phases (day 24) of joint inflammation. C3-deficient mice showed no differences in technetium uptake or histological scoring. TLR2-deficient mice also showed a significant decrease in lymph node cell proliferation in response to zymosan and a lower IgG antibody response to zymosan at day 25 in comparison with wild-type controls, indicating that TLR2 signalling has a role in the development of acquired immune responses to zymosan. Although laminarin, a soluble β-glucan, was able to significantly inhibit zymosan uptake by macrophages *in vitro*, it had no effect on ZIA *in vivo*. These results show that ZIA is more prolonged than was originally described and involves both the innate and acquired immune pathways. C3 does not seem to have a major role in this model of joint inflammation.

## Introduction

Zymosan, a polysaccharide from the cell wall of *Saccharomyces cerevisiae*, is composed primarily of glucan and mannan residues [1]. *In vitro*, it has served as a model for the study of innate immune responses, because it is capable of stimulating inflammatory cytokine production [2] and can activate complement in the absence of immunoglobulins [3]. Zymosan is recognized and phagocytosed principally by monocytes and macrophages and leads to cellular activation [4]. Zymosan-induced arthritis (ZIA) in mice was first described by Keystone in 1977 [5]. Arthritis was induced by intra-articular injection of zymosan and was thought to be mediated by activation of the alternative pathway of complement and the release of lysosomal hydrolases from activated macrophages [6].

The recent discovery of pattern recognition receptors and their role in innate immunity has led to a re-evaluation of our concepts of zymosan-induced inflammation. Toll-like receptors (TLRs) are a family of type 1 transmembrane proteins that consists of an extracellular leucine-rich repeat domain and a cytoplasmic domain homologous to the cytoplasmic domain of the human interleukin 1 (IL-1) receptor [7]. The ligands of TLR2 include lipopeptides and peptidoglycan [8,9], and TLR2 is a receptor for zymosan, acting in collaboration with CD14 and TLR6 [2,10]. Ligand binding to TLRs induces the activation of NF-κB and the production of the inflammatory cytokines IL-1, IL-6, IL-8, and IL-18 as well as the expression of the co-stimulatory molecule B7.1 [7]. Additionally, zymosan is able to induce maturation of dendritic cells *in vitro *and to stimulate their production of IL-2 [11,12], providing evidence for a link between the innate and the adaptive immune responses.

The inflammatory response triggered by zymosan is linked to its phagocytosis, a process that is mediated by a set of different receptors from the TLRs. The non-opsonic recognition of zymosan by macrophages is mediated by Dectin-1. Dectin-1 is a type 2 membrane receptor with an extracellular C-type lectin-like domain fold and a cytoplasmic immunoreceptor tyrosine-based activation motif [13] and is expressed on macrophages, dendritic cells and neutrophils [14-16]. Dectin-1 mediates the binding of *Saccharomyces cerevisiae *and *Candida albicans *in a β-glucan-dependent manner and may also have a pro-inflammatory function [17].

In the light of the above findings, we have re-investigated ZIA to elucidate the roles of the innate and adaptive immune responses in this model and to compare the effects of TLR2 deficiency and complement C3 deficiency. The role of Dectin-1 in zymosan-induced inflammation was also investigated. Our results indicate that TLR2 is the major pathway of pro-inflammatory signalling in ZIA and is necessary for the development of specific immune responses to zymosan.

## Materials and methods

### Animals

C3-deficient mice (C3^-/-^) on a C57bl/6 background were generated by Professor M Botto [18]. TLR2-deficient mice (TLR2^-/-^) on a C57bl/6 background were provided by Dr Kiyoshi Takeda (Department of Host Defense, Research Institute for Microbial Diseases, Osaka University) [19]. Wild-type (WT) C57bl/6 mice were purchased from Charles River (L'Arbresle, France). All mice were bred in our animal house facility. Double knockout and double WT mice were generated by mating TLR2^-/- ^and C3^-/- ^mice. The genotypes of all mice used were confirmed by polymerase chain reaction analysis of genomic DNA extracted from mice tails. The primer sequences used were as follows: TLR2 sense, 5' -GTTCTCCCAGCATTTAAAATCATT-3' ; TLR2 antisense, 5' -GTCTCCAGTTTGGGAAAAGAACC-3' ; TLR2 NEO antisense, 5' -CGACACAGCTGCGCAAGCAAC-3' ; C3 sense, 5' -CTTCATAGACTGCTGCAACCA-3' ; C3 antisense, 5' -AACCAGCTCTGTGGGAAGTG-3' ; C3 NEO antisense, 5' -AAGGGACTGGCTGCTATTGG-3'.

### Induction of ZIA

Zymosan A from *Saccharomyces cerevisiae *(Sigma, St Louis, MO, USA) (300 mg) was resuspended in 10 ml of endotoxin-free saline, boiled and homogenized by sonic emulsification. The suspension was autoclaved and stored in aliquots at -20°C. Arthritis was induced by intra-articular injection of 180 μg (6 μl) of zymosan through the suprapatellar ligament into the joint cavity. In specified experiments, the contralateral knee was injected with an equal amount of sterile saline (6 μl) as control.

Laminarin was co-injected at a dose of either 500 μg or 100 μg together with 180 μg of zymosan into the knee joint.

Approval was obtained from the local animal health committee for these experiments.

### Isotopic quantification of joint inflammation *in vivo*

Joint inflammation was measured by ^99m^Tc uptake in the knee joint as described [20]. Mice were sedated by the intra-peritoneal administration of sodium pentobarbital (50 mg/kg) and then injected subcutaneously in the neck region with 10 μCi of ^99m^Tc. The accumulation of the isotope in the knee was determined by external gamma-counting after 15 min. The ratio of ^99m^Tc uptake in the inflamed arthritic knee to ^99m^Tc uptake in the contralateral control knee was calculated. A ratio higher than 1.1:1 indicated joint inflammation.

### Histological grading of arthritis

Mice were killed at day 8 and at day 25. Knees were dissected and fixed for 2 weeks in 10% buffered formalin. Fixed tissues were decalcified for 2 weeks in 15% EDTA, dehydrated and embedded in paraffin. Sagittal sections (5 μm) of the whole knee joint were stained with safranin-O and counterstained with fast green/iron hematoxylin. Histological sections were graded by two observers unaware of animal genotype or treatment. Synovial cell infiltrate and exudate were scored from 0 (no cells) to 6 (maximum number of inflammatory cells). Cartilage proteoglycan depletion (damage), reflected by a loss of safranin-O staining intensity, was scored on a scale from 0 (fully stained cartilage) to 6 (totally unstained cartilage) in proportion to severity. For each histopathological measure the score (mean ± SEM) of all slides was calculated.

### T cell proliferation assay

Mice were killed in accordance with the experimental protocol. Inguinal lymph nodes were removed and single-cell suspensions were incubated in RPMI supplemented with 2-mercaptoethanol, penicillin, streptomycin and 1% autologous serum. Lymph node cells (LNC; 4 × 10^5 ^per 200 μl per well) were plated in 96-well flat-bottomed plates and stimulated with zymosan at specified concentrations. Concanavalin A at 4 μg/ml was used as non-specific mitogen.

The cells were incubated for 48 hours at 37°C in 5% CO_2_, then [^3^H]thymidine (1 μCi per well) was added to the cultures for 18 hours. The cells were harvested, and [^3^H]thymidine uptake was measured with a beta scintillation counter.

### Determination of interferon-γ production *in vitro*

Culture supernatants from LNC cultured with or without 4 μg/ml zymosan were harvested after 72 hours for determination of interferon (IFN)-γ levels. Quantification of cytokine production was performed with an enzyme-linked immunosorbent assay (ELISA) kit specific for murine IFN-γ (Amersham Pharmacia, Dubendorf, Switzerland).

### TLR2 immunohistochemistry

Immunohistochemistry was performed with affinity purified anti-mouse TLR2 antibody (clone 6C2; eBioscience, San Diego, CA, USA). Specificity of the antibody was tested on bone marrow cells derived from c57bl/6 TLR2^+/+ ^and TLR2^-/- ^mice.

Dissected knees were embedded in Tissue-Tek OCT, then immediately frozen in precooled hexane and stored at -70°C until use. Sections 7 μm thick were cut on a motor-driven Leica cryostat with a retraction microtome and a tungsten carbide knife at a cabinet temperature of -25°C and mounted on Menzel Super Frost Color glass slides.

### Phagocytosis assay

RAW 264.7 cells (5 × 10^5 ^to 10^6 ^per chamber) were plated on a Lab-Tek II Chamber Slide system (Nalge Nunc International). After adherence, cells were either preincubated with 100 or 500 μg/ml laminarin [21] for 20 min followed by the addition of 25 zymosan particles per cell, or laminarin was co-administrated with zymosan. After incubation for 3 hours at 37°C in 5% CO_2_, cells were washed twice with PBS and fixed for 10 min in acetone. Cell-bound and phagocytosed particles were stained by periodic acid Schiff, a stain specific for insoluble glucose polymers, and quantified by light microscopy.

### Quantification of IgG levels

Serum levels of total IgG were quantified with ELISA. In brief, rabbit anti-mouse IgG (Dako, Carpinteria, CA, USA) was coated on 96-well plates (Nunc, Roskilde, Denmark). Murine sera from naive and ZIA mice (dilution 1:100,000) were added and incubated for 2 hours. Secondary alkaline-phosphatase-linked anti-mouse IgG (Sigma, Buchs, Switzerland) was added and *p*-nitrophenyl phosphate (Sigma, Buchs, Switzerland) completed the reaction.

Serum levels of specific anti-zymosan IgG were also quantified by ELISA. Zymosan particles at 1 mg/ml were coated on 96-well plates and murine sera (dilution 1:100) were added and incubated for 2 hours. The reaction was developed as previously described.

### Statistical analysis

The Wilcoxon rank sum test for unpaired variables (two-tailed) was used to compare differences between groups. The unpaired Student *t*-test was used to compare the groups with normally distributed values. A level of *P *< 0.05 was considered statistically significant.

## Results

### Zymosan-mediated inflammation in the knee joint is biphasic

**Figure 1:**
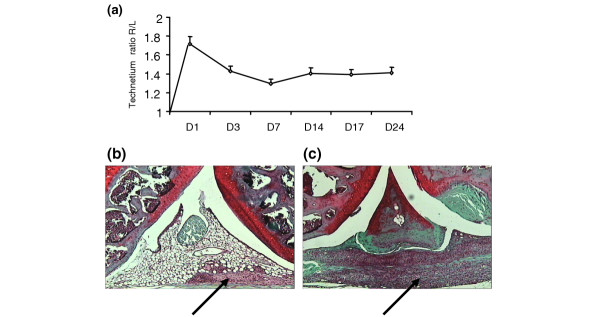
Biphasic kinetic of inflammation in zymosan-induced arthritis (ZIA). **(a) **^99m^Tc uptake measurement shows a biphasic course of inflammation in wild-type (WT) mice. Zymosan was injected into the right knee and PBS was injected into the contralateral control knee. The values obtained correspond to the ratio between the right and left knee (*n *= 15). The severity of histological signs of arthritis was assessed by scoring synovial thickness and cartilage destruction on a scale from 0 to 6. Results are expressed as means ± SEM. **(b) **WT mice with ZIA showed mild inflammation at day 8 as judged by cellular infiltration (histological score 1.00 ± 0.32) and cartilage destruction (0.7 ± 0.2; *n *= 10), which became more severe at day 25. **(c) **There was an increase in cellular infiltration (2.5 ± 0.37) but cartilage destruction remained slight (0.71 ± 0.21; *n *= 10). The arrows in (b) and (c) indicate inflammatory infiltrate in the synovial membrane.

In experiments on WT C57bl/6 mice, we observed a biphasic course of inflammation, with an initial peak of ^99m^Tc uptake at day 1 (1.71 ± 0.08), followed by a decrease to a trough value at day 7 (1.29 ± 0.05) and a secondary increase in uptake at day 14. Inflammation measured by ^99m^Tc uptake persisted up to day 25 (1.40 ± 0.06) (Fig. 1a).

Histological assessment of the mice at day 8 showed a low score for cellular infiltration (1.00 ± 0.32) and for cartilage destruction (0.7 ± 0.2) (Fig. 1b), whereas scoring at day 25 was characterized by an increase in cellular infiltration (2.5 ± 0.37) while cartilage destruction remained low (0.71 ± 0.24) (Fig. 1c).

### Histology and immune responses at day 25 of ZIA

**Figure 2:**
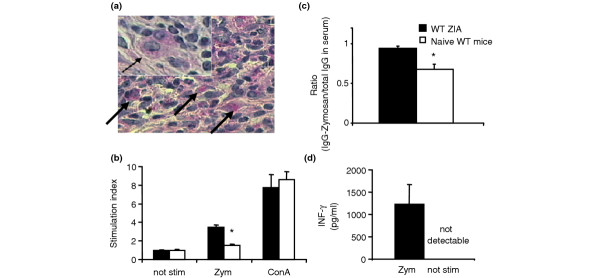
Local and immune responses to zymosan at day 25. **(a) **Periodic acid Schiff staining shows the persistence of zymosan particles (arrow) within the synovial membrane in zymosan-induced arthritis (ZIA). Original magnification ×100. **(b) ***In vitro *lymph node mononuclear cell proliferation in response to zymosan at day 25 of ZIA and in naive mice. Single-cell suspensions were incubated with 4 μg/ml zymosan. Concanavalin A (ConA) was used as a non-specific mitogen. **(c) **Antibody production against zymosan in zymosan-treated mice compared with naive mice measured by enzyme-linked immunosorbent assay (ELISA). The results are expressed as a ratio of the amount of zymosan-specific IgGs (in arbitrary units) in murine serum to total IgGs (also in arbitrary units) (*P *< 0.05). **(d) **Interferon-γ (IFN-γ) production by zymosan-stimulated and unstimulated lymph node cells of WT ZIA and naive mice in culture. IFN-γ was measured by specific ELISA. n.d., not detectable.

To determine whether zymosan particles persisted in the joint at day 25, periodic acid Schiff staining was performed on joint tissues obtained at day 25 and showed persistence of zymosan particles in the synovial membrane of mice injected with zymosan (Fig. 2a).

To verify that joint inflammation was associated with the development of specific immune responses to zymosan, we assessed both humoral and cellular responses in WT mice. Proliferation of LNC in response to zymosan was significantly increased in day 25 WT ZIA mice compared with LNC of naive mice (3.5 stimulation index in ZIA WT mice versus 1.5 in naive mice; *P *< 0.001), whereas mitogenic response to the non-specific mitogen concanavalin A at 4 μg/ml showed no difference between groups (Fig. 2b). No difference in proliferation in response to zymosan was observed between ZIA and naive mice at day 8 (data not shown).

The humoral response to zymosan was measured by ELISA. In arthritic mice, the serum levels of anti-zymosan IgG antibodies were significantly increased at day 25 in comparison with those in untreated naive mice (antibody ratio for WT = 0.944 versus naive = 0.677; *P *< 0.02) (Fig. 2c).

In addition, *in vitro *stimulation of WT ZIA LNC with zymosan at 4 μg/ml induced the secretion of IFN-γ at 1200 pg/ml, whereas unstimulated LNC produced undetectable levels of IFN-γ (Fig. 2d).

### Synovial expression of TLR2 and its role in ZIA

**Figure 3:**
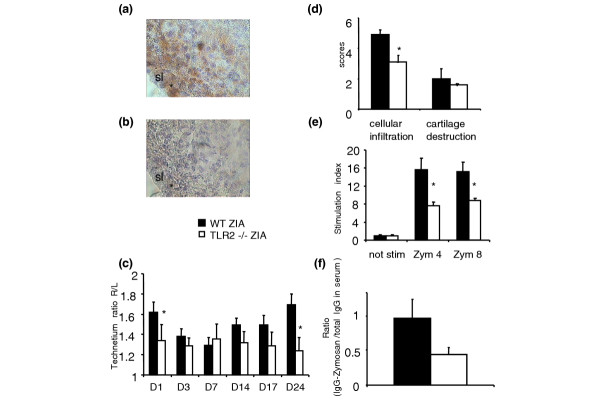
TLR2 mediates an inflammatory response in zymosan-induced arthritis (ZIA). **(a) **Immunohistochemistry of TLR2 expression in synovial membrane sections of WT mice (day 25 ZIA) showed staining in the sublining (sl) of synovial membrane, whereas inflammatory lymphocytes were not stained. **(b) **Negative control was performed with 0.5% bovine serum albumin. (100× magnification). **(c) **^99m^Tc uptake measurement showed an attenuation of the inflammatory response at days 1 and 25 (*P *< 0.05) in TLR2^-/- ^ZIA mice (*n *= 5) compared with WT ZIA mice (*n *= 5). The technetium ratio was measured as detailed in the Materials and methods section. **(d) **Histological scoring at day 25 of ZIA showed a significant decrease in cell infiltration in TLR2^-/- ^mice (*P *< 0.02) compared with control mice, whereas cartilage destruction was similar in both groups. **(e) **Lymph node cell proliferation to 4 and 8 μg/ml zymosan (Zym 4 and Zym 8, respectively). **(f) **Antibody production against zymosan in zymosan-treated TLR2^+/+ ^and TLR2^-/- ^mice.

We wished next to evaluate whether TLR2 might have a role in the recognition of zymosan *in vivo *and in mediating inflammation in ZIA. Specific antibody for TLR2 was used to stain synovium from WT mice that had developed ZIA at day 25. Figure 3a shows a representative example of the distribution of TLR2 expression in the synovial cell lining. Control antibody staining was negative (Fig. 3b). Antibody specificity was confirmed by a lack of staining in TLR2^-/- ^mice (data not shown).

To explore whether the deficiency of TLR2 had an effect on the course of ZIA, we measured knee joint inflammation in TLR2^+/+ ^and TLR2^-/- ^mice by ^99m^Tc uptake at different time points up to day 24 (Fig. 3c). In two independent experiments we observed an attenuation of inflammation in TLR2^-/- ^mice at days 1, 3, 14, 17 and 24, although only the decrease observed at days 1 and 24 reached statistical significance (*P *< 0.05).

### TLR2 deficiency ameliorates histological features of ZIA

We compared the histological features of arthritic knee joints from TLR2^+/+ ^and TLR2^-/- ^mice (Fig. 3d). In both groups, arthritis was histologically present in all knees that had been injected with zymosan. In TLR2^+/+ ^mice, on day 25 of ZIA, the synovial membrane was thickened, mainly as a result of invasion by inflammatory cells (see Fig. 1c). In TLR2^-/- ^mice, synovial infiltrate was significantly decreased in comparison with TLR2^+/+ ^mice (4.9 ± 0.33 in TLR2^+/+ ^mice [*n *= 15] versus 3.1 ± 0.67 in TLR2^-/- ^mice [*n *= 12] on day 25 after arthritis onset; *P *< 0.045).

TLR2^-/- ^mice showed no difference from WT mice in terms of cartilage destruction, as assessed by the loss of safranin-O staining at day 25 (Fig. 3d).

### Effect of TLR2 deficiency on cellular responses

The role of TLR2 on the cellular response to zymosan was examined by isolating LNC from ZIA mice. The proliferation of LNC induced by zymosan was significantly lower in cells isolated from TLR2^-/- ^mice than in TLR2^+/+ ^mice. A significant difference was found at both concentrations of zymosan studied (4 and 8 μg/ml; both *P *< 0.05) (Fig. 3e). No differences were observed in proliferation stimulated by the non-specific mitogen concanavalin A (data not shown).

The serum levels of anti-zymosan IgG antibodies, measured by ELISA, were decreased by 50% in TLR2^-/- ^mice at day 25 in comparison with the serum levels in controls (antibody ratio for WT = 1.00 versus TLR2^-/- ^= 0.51, *P *= 0.047) (Fig. 3f).

### Lack of effect of C3 on inflammation in ZIA

The availability of C3-deficient mice in a C57bl/6 background allowed us to reassess the role of C3 in ZIA. No effect, either in ^99m^Tc uptake or in histological scoring, was observed in C3-deficient (*n *= 25) mice in comparison with WT mice (*n *= 25). In addition, humoral and cellular responses were similar in C3^-/- ^and C3^+/+ ^mice (data not shown).

**Figure 4:**
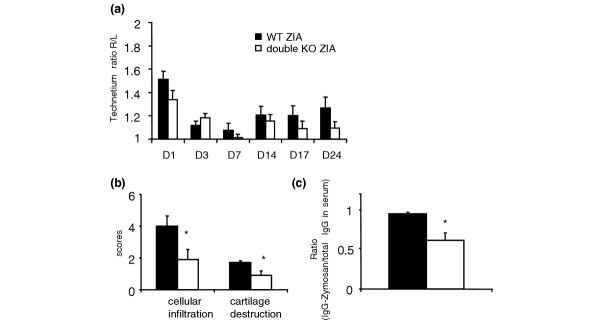
C3 has no role in mediating inflammatory responses in zymosan-induced arthritis. **(a) **^99m^Tc uptake measurement showed an attenuation of inflammation at early and late time points in TLR2/C3 double-deficient (KO) mice (*n *= 5) compared with double wild-type (WT) mice (*n *= 5) but did not reach statistical significance. **(b) **Histological scoring showed a significant decrease in both cell infiltration (*P *< 0.05) and cartilage destruction (*P *< 0.05) in TLR2/C-3 double-deficient mice (*n *= 5) compared with littermate control (*n *= 5). **(c) **Production of specific anti-zymosan IgGs was reduced in double-deficient mice in comparision with double WT littermates. Values correspond to the ratio of zymosan-specific IgG to total IgG mentioned in Fig. 2c.

Generation of TLR2/C3 double-deficient mice gave similar responses as TLR2^-/- ^mice, excluding a synergistic effect of double deficiency and confirming no role for the alternative pathway component of the complement cascade (Fig. 4a). Histological scoring showed the presence of arthritis in both groups of animals. In TLR2/C-3 double-deficient mice, synovial infiltrate was significantly decreased in comparison with control (4.0 ± 0.65 in control mice [*n *= 5] versus 1.9 ± 0.62 in TLR2/C-3 double-deficient mice [*n *= 5] on day 25 after arthritis onset; *P *< 0.05) (Fig. 4b).

TLR2/C-3 double-deficient mice also showed a significantly decreased cartilage destruction in comparison with WT mice at day 25 (1.7 ± 0.12 in control mice [*n *= 5] versus 0.9 ± 0.29 in TLR2/C-3 double-deficient mice [*n *= 5]; *P *< 0.05) (Fig. 4b).

Stimulation of LNC with zymosan *in vitro *showed a significant decrease of stimulation in double-deficient mice compared with WT littermates, similar to that observed in TLR2^-/- ^mice (data not shown).

In addition, a decreased production of zymosan-specific IgGs was observed in the double-deficient mice (ratio for WT = 0.944 versus double knockout = 0.616; *P *< 0.05) (Fig. 4c).

### Dectin-1 has a minor role in inflammation in ZIA

**Figure 5:**
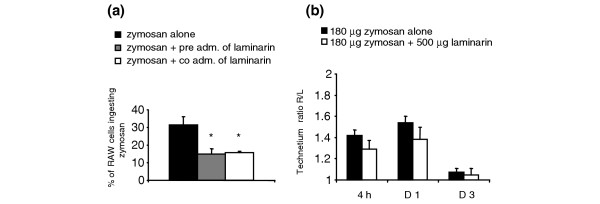
Dectin-1 inhibition *in vitro *and *in vivo*. **(a) **In a phagocytosis assay, 2.5 × 10^6 ^particles of zymosan were incubated with 10 × 10^5 ^RAW 264.7 cells. Laminarin was administered either before (pre adm.) or at the same time as (co adm.) the zymosan particles. Incubation of zymosan particles was performed for 4 hours at 37°C. Laminarin decreased the amount of zymosan bound to and phagocytosed by RAW 264.7 cells by 50% in comparison with a control to which no laminarin had been added (*P *< 0.05). **(b) **Laminarin (500 μg) and zymosan (180 μg) in a final volume of 15 μl were co-injected into the right knee joint of C57bl/6 mice (*n *= 6). Phosphate-buffered saline (15 μl) was injected into the contralateral knee. ^99m^Tc uptake was measured up to day 7. Comparison of ^99m^Tc uptake with control mice (*n *= 8), which received zymosan alone in the right knee, showed an attenuation of inflammation at 4 and 24 hours in the laminarin-treated mice, but did not reach statistical significance. After 72 hours the values were similar.

The identification of the β-glucan receptor Dectin-1 and its ability to bind zymosan particles *in vitro *stimulated us to study the role of Dectin-1 *in vivo *in ZIA. *In vitro *blockade of the Dectin-1 receptor by laminarin led to a 50% decrease in a phagocytosis assay with RAW 264.7 cells. This decrease was not dependent on the time of administration of laminarin, because it was not modified by preincubation or co-incubation with zymosan particles (Fig. 5a).

Co-administration of laminarin and zymosan in the knee joint of C57bl/6 mice showed a trend to a decrease of ^99m^Tc uptake in the early phase of inflammation in a laminarin-treated knee, compared with an untreated knee, at 4 hours and 1 day after administration, but did not reach statistical significance (Fig. 5b).

## Discussion

For more than 50 years zymosan has been a tool in the study of microbial recognition by the innate immune system. The mechanisms mediating the recognition and phagocytosis of zymosan *in vivo *are complex. Phagocytes, including monocytes, macrophages and dendritic cells, express receptors such as the TLRs, complement receptor 3, scavenger receptors (such as acetylated LDL receptors) and Dectin-1 [22-24], which have all been implicated in the cellular response to zymosan [25]. In addition, zymosan is capable of activating the alternative pathway of complement through C3 [3], which may serve to amplify the inflammatory response.

To elucidate how zymosan induces inflammation *in vivo*, we re-investigated the ZIA model that was first studied in the 1970s. This model has been often used as a tool to dissect non-immune mechanisms of joint inflammation [26-28]. In our experiments we observed that ZIA was not as short lived as originally described. Arthritis persisted beyond day 14 and in fact beyond day 25. After an initial peak of inflammation at about day 3, inflammation subsided by day 7. Subsequently, inflammation returned to levels that could be as high (as measured by ^99m^Tc uptake) as the initial peak, suggesting that ZIA has early and late phases. Histologically, the joint inflammation was characterized by mononuclear cell infiltration in the sublining layer and hypertrophy of the lining layer as well as cartilage damage. Histological changes were milder at day 8 than at day 25. Zymosan particles were present in the synovium at day 25.

We then investigated the role of TLR2 in ZIA because the macrophage inflammatory response to zymosan depends largely on its recognition by a heterodimer of TLR2 and TLR6 [2,10]. In TLR2^-/- ^mice there was a significant attenuation of the early and late inflammatory phases of ZIA, indicating that a ligand that activates the innate immune response through TLR2 can lead to a chronic local inflammatory reaction.

In the absence of TLR2, joint inflammation was not totally blocked. This would suggest that, *in vivo*, the inflammatory response to zymosan is not dependent on TLR2 signalling alone and that receptors other than TLR2 might have a role. This is supported by the observation that inhibition of TLR2 and MyD88 by dominant-negative mutants blocked pro-inflammatory signalling but not zymosan uptake *in vitro*. Recent data have shown that Dectin-1 and SIGNR1 [29] on macrophages and pentraxin-3, an opsonin for the recognition of zymosan by Dectin-1, are involved in zymosan recognition and internalization [30]. We therefore investigated the role of Dectin-1 by using the β-glucan laminarin as a competitive inhibitor of zymosan [16]. We confirmed that laminarin inhibited zymosan uptake by RAW 264.7 cells and did not observe any difference in the blocking capacity of laminarin, whether administered before or at the same time as zymosan. In both cases and at two different concentrations, we observed a 50% decrease in cell-bound zymosan particles. On the basis of these results, we proceeded to assess the effect of laminarin on ZIA. Although there was a trend towards reduced ^99m^Tc uptake in the treated animals, this was not statistically significant. It is possible that 50% inhibition of zymosan phagocytosis is insufficient to modulate inflammatory signalling through TLR2. Furthermore, a redundancy in the multiple mechanisms that mediate zymosan phagocytosis could also explain the lack of effect of laminarin inhibition *in vivo *[31].

The biphasic course of ZIA and its modulation by TLR2 led us to study the acquired immune response to zymosan and the effects of TLR2 deficiency on it. We compared the cellular proliferative and antibody responses to zymosan in WT and TLR2^-/- ^mice at day 25. First, we were able to detect zymosan-induced lymphocyte proliferation and enhanced IFN-γ production in the draining LNC of mice with ZIA, and second, this was accompanied by the formation of a zymosan-specific IgG. In TLR2^-/- ^animals, the proliferative response was blunted and only reached 50% of that observed in WT ZIA animals. There was also a significant decrease in the zymosan-specific IgG response, which was about 50% lower than in WT mice. At day 8 we did not observe any difference between ZIA and naive WT mice in their proliferative response to zymosan (data not shown). These findings suggest that inflammation in the later phase of ZIA is paralleled by the development of acquired immune response to zymosan. The finding that zymosan particles persisted in the joint even at day 25 suggests that they could become a target for specific immune responses. The decrease in acquired immune responses in TLR2^-/- ^mice might be a result of the decreased antigen presentation efficiency of dendritic cells in the absence of TLR2 [32] or the lack of co-stimulatory signals through TLR2 expressed on activated T cells [33].

A significant role for the alternative pathway of complement in this model of inflammation was excluded by the phenotype observed in C3^-/- ^mice. Both phases of ZIA were comparable to that observed in WT controls. Mice with combined deletions of the C3 and the TLR2 genes did not show a significant decrease in ^99m^Tc uptake in comparison with TLR2^-/- ^mice. Histologically, we observed a significant decrease in cartilage damage in mice with the combined deficiency of TLR2 and C3, which did not occur in C3^-/- ^mice. We interpret this effect to be due in principle to the lack of TLR2, because TLR2^-/- ^mice also showed a diminished cartilage score (although it did not reach statistical significance). Combined with recent data showing that phagocytosis of zymosan is not mediated by complement receptor 3 [16], complement activation does not seem to contribute to zymosan-induced joint inflammation *in vivo*.

The expression of TLR2 in arthritic synovium from WT mice in the ZIA model and the modulation of joint inflammation in TLR2^-/- ^animals show that TLR2 may have a general role in amplifying local inflammation. TLR2 has been shown to be expressed on neutrophils and lymphocytes as well as macrophages, and they all are participants in the inflammatory process in this model. The data in human arthritis would also support such a role for TLR2. Increased expression of TLR2 in synovial lining layer and by CD16^+ ^peripheral blood mononuclear cells in RA indicate that its expression is upregulated during chronic inflammation [34]. TLR2 is also expressed on RA synovial fibroblasts, and incubation of cultured RA synovial fibroblasts with pro-inflammatory cytokines increases levels of TLR2 mRNA [35]. Although the precise role of TLR signalling in RA is unclear at present, increased TLR2 expression might modulate synovial inflammation if endogenous or exogenous TLR2 ligands gain access to the joint, thus amplifying specific and innate immune pathways of synovial inflammation. Furthermore, our results provide a model by which stimulation of the innate immune response can lead to chronic inflammation in the joint. These pathways may be of relevance to the development of reactive arthritis in man.

## Conclusion

The results of the present study indicate that the biphasic joint inflammation in ZIA is mediated primarily by activation of the innate immune system. In the early phase of arthritis TLR2 plays a vital role, and in the later phase the development of a secondary immune response to zymosan may contribute to joint inflammation. Innate immune responses may be important amplificatory pathways of joint inflammation in man.

## Abbreviations

ELISA = enzyme-linked immunosorbent assay; IFN = interferon; IL = interleukin; LNC = lymph node cells; TLR = Toll-like receptor; WT = wild-type; ZIA = zymosan-induced arthritis.

## Competing interests

The author(s) declare that they have no competing interests.

## Authors' contributions

MF contributed to breeding and genotyping, performed technetium uptake measurements and immunoassays, and participated in coordination of the study. DT participated in technetium uptake measurements. VC performed histological stainings and scoring. NB performed statistical analysis and participated in the design of the study. AS conceived of the study, participated in its design and coordination, and helped to draft the manuscript. All authors read and approved the final manuscript.
